# The Shift from Multiport to Single Port Increases the Amount of Bleeding in Laparoscopic Major Hepatectomy

**DOI:** 10.3390/jcm10030374

**Published:** 2021-01-20

**Authors:** Christof Mittermair, Michael Weiss, Jan Schirnhofer, Eberhard Brunner, Katharina Fischer, Christian Obrist, Michael de Cillia, Vanessa Kemmetinger, Emanuel Gollegger, Tobias Hell, Helmut Weiss

**Affiliations:** 1 Surgical Department, St John of God Hospital, Teaching Hospital of the Paracelsus Medical University Salzburg, Kajetanerplatz 1, 5010 Salzburg, Austria; christof.mittermair@bbsalz.at (C.M.); michael.weiss1@icloud.com (M.W.); jan.schirnhofer@bbsalz.at (J.S.); eberhard.brunner@bbsalz.at (E.B.); katharina.fischer@bbsalz.at (K.F.); christian.obrist@bbsalz.at (C.O.); michael.decillia@bbsalz.at (M.d.C.); vanessa.kemmetinger@bbsalz.at (V.K.); praxis@dr-gollegger.at (E.G.); 2 Department of Mathematics, University of Innsbruck, Technikerstrasse 13, 6020 Innsbruck, Austria; tobias.hell@uibk.ac.at

**Keywords:** hepatectomy, single-port laparoscopy, radiofrequency pre-coagulation

## Abstract

Background: Bleeding is a negative outcome predictor in liver surgery. Reduction in the abdominal wall trauma in major hepatectomy is challenging but might offer possible benefits for the patient. This study was conducted to assess hemostasis techniques in single-port major hepatectomies (SP-MajH) as compared to multiport major hepatectomies (MP-MajH). Methods: The non-randomized study comprised 34 SP-MajH in selected patients; 14 MP-MajH served as the control group. Intraoperative blood loss and number of blood units transfused served as the primary endpoints. Secondary endpoints were complications and oncologic five-year outcome. Results: All resections were completed without converting to open surgery. Time for hepatectomy did not differ between SP-MajH and MP-MajH. Blood loss and number of patients with blood loss > 25 mL were significantly larger in MP-MajH (*p* = 0.001). In contrast, bleeding control was more difficult in SP-MajH, resulting in more transfusions (*p* = 0.008). One intestinal laceration (SP-MajH) accounted for the only intraoperative complication; 90-day mortality was zero. Postoperative complications were noted in total in 20.6% and 21.4% of patients for SP-MajH and MP-MajH, respectively. No incisional hernia occurred. During a median oncologic follow-up at 61 and 56 months (SP-MajH and MP-MajH), no local tumor recurrence was observed. Conclusions: SP-MajH requires sophisticated techniques to ensure operative safety. Substantial blood loss requiring transfusion is more likely to occur in SP-MajH than in MP-MajH.

## 1. Introduction

The scientifically proven benefits of minimally invasive liver surgery justify the effort to further develop the technique [1]. Single-port laparoscopy (SP) is regarded as the most ambitious approach to minimize abdominal wall trauma in hepatic resection. The successful concept of aligning the entire procedure only via the incision that is necessary to retrieve the specimen has been scientifically evaluated in various organ systems such as colorectal and biliary surgeries [2,3]. In addition, the possibility to avoid vascular injury in portal hypertension by reducing the number of incisions and to alleviate repeated interventions for hepatic metastasis by preventing the formation of adhesions can be considered potential benefits in the group of these patients.

As compared to multiport laparoscopy, SP liver surgery is advantageous in terms of reduced blood loss while providing the same effectiveness and optimal patient safety and recovery [4].

Due to the technical obstacles encountered, such as the combination of different instruments that have to be delivered simultaneously through one single fulcrum to expose the operation field and to provide suction, flushing, coagulation or clipping, SP major hepatic resection (SP-MajH) is not performed at most liver centers. In particular, intraoperative bleeding control is at the center of interest as blood loss is one of the main adverse prognostic parameters for short-term and long-term outcomes [5,6]. Pre-coagulation by means of intraoperative radiofrequency-assisted transection of the hepatic parenchyma allows for ideal blood vessel sealing, without an increase in biliary complications [7]. We were previously able to demonstrate that SP minor liver resection benefits from the possibility to use inline pre-coagulation [8].

The study was conducted to evaluate the currently largest series of SP-MajH compared to multiport laparoscopic major hepatectomies (MP-MajH) with regard to bleeding control.

## 2. Materials and Methods

From September 2008 to November 2018, a total of 96 SP liver resections with inline pre-coagulation were performed at the surgical department of the St John of God Hospital, Salzburg, Austria. This accounts for 22.4% of all hepatic resections (*n* = 429) and 1.9% of the SP patient cohort (*n* = 5095) in that period of time.

Procedures were categorized as minor and major liver resection according to the 2nd International Consensus Conference for Laparoscopic Liver Surgery [9]. Major liver resection was defined as removal of >2 Couinaud segments or resections including at least one of the segments I, IVa, VII or VIII. Difficulty index, including tumor location, extent of liver resection, tumor size, proximity to major vessels and liver function, was calculated as proposed by Ban and colleagues [10].

A total of 34 single-port laparoscopic major hepatectomies (SP-MajH) were consecutively performed during the study period (study population).

At the same time, 14 multiport major hepatectomies (MP-MajH) with the identical procedural strategy were also performed (29.2% of all minimally invasive major liver resections) solely because of a lack of resources for SP surgery. These patients served as the control group in the comparison between SP and MP major hepatic resections.

All types of benign and malignant liver diseases requiring surgical treatment were considered for enrolment in the study. Prior abdominal surgery, higher age, obesity or unfavorable American Society of Anesthesiologists (ASA) scoring were not regarded as a contraindication for the SIL approach. Exclusion criteria for minimally invasive surgery were defined as follows: Child–Pugh B or C cirrhosis, future liver remnant volume <50%, tumor growth in close approximation to vital pedicles and as the only relative contraindication for SIL denial at the surgeon’s discretion.

Preoperative routine testing, including CT and MRT, was performed according to international guidelines. Indication for the operation was confirmed by the local tumor board in all malignant cases. Informed consent was obtained from all patients following the standards of the Helsinki Declaration. The SP technique was approved by the local ethics committee. All SP procedures were performed by surgeons trained in both hepato-bilio-pancreatic surgery and advanced SP.

### 2.1. Procedure

**Figure 1:**
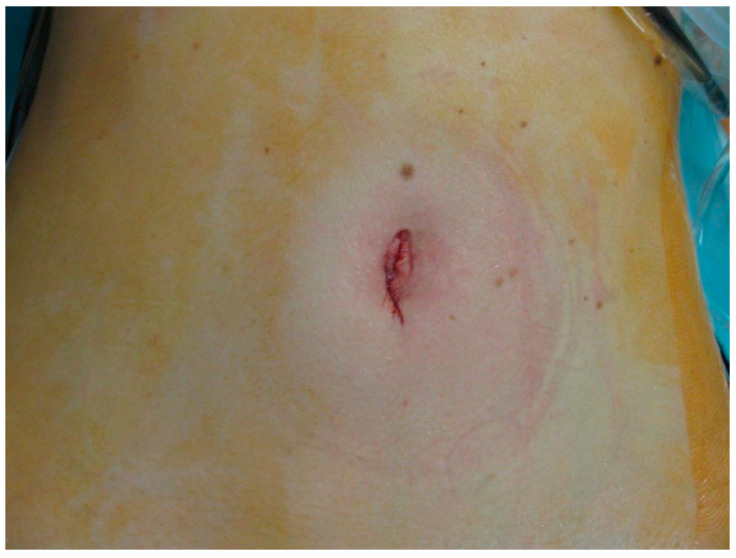
Image of the abdominal scar following single-port (SP) major hepatectomy (resections of segments VII and VIII).

Patients were placed in the reverse Trendelenburg position (20° head up) with their legs apart (French position). For posterior or right lateral resections, a 45° left lateral decubitus position alleviated exposure. Single-port access was obtained through the umbilicus, pre-existing scars in the upper abdomen (midline or subcostal) or a right subcostal incision in the midclavicular line (Figure 1).

The GelPort™ (*n* = 38; Applied Medical, Rancho Santa Margarita, CA, USA) and the OctoPort™ (*n* = 10; DalimSurgNET, Frankenman Group, Seoul, Korea) in combination with the AirSeal™ System (SurgiQuest, Milford, CT, USA) were used to maintain the pneumoperitoneum at 12 mmHg.

A 10 mm, 30° extra-long optic and at least one articulating grasper were used throughout all procedures. Suction or retraction was controlled by the surgical assistant guiding the instrument through the same port. Suspending sutures for the triangular ligament were placed as needed. Laparoscopic ultrasound ensured the proper resection margin. The Pringle maneuver was not used routinely.

Exposure of central pedicles was mastered by means of bipolar cautery and clips. Prior to parenchymal transection, inline pre-coagulation was primarily accomplished with the HABIB 4X bipolar resection device (RITA Medical Systems, AngioDynamics, Latham, NY, USA). Liver packing was performed to prevent thermal injury to surrounding organs or the diaphragm. Parenchymal transection was subsequently performed with monopolar scissors or the LigaSureV™ (Medtronic, Dublin, Ireland) device. The CUSA (Cavitron Ultrasonic Surgical Aspirator; Medtronic, Dublin, Ireland), hemoclips, parenchymal sutures or vascular staplers served as second-line devices as needed.

Specimen retrieval was realized with a tear-proof bag (Espiner Medical, Clevedon, UK), allowing tissue compression to minimize the incisional length and guarantee correct pathohistological assessment.

Hemostatic matrix foam (Flowseal™, Baxter, Deerfield, IL, USA) or TachoSil™ fibrin sealant patch (Baxter, Deerfield, IL, USA) were applied at the surgeon’s discretion. Wound closure was performed with monofilament, non-reabsorbing fascial running sutures and intra-cuticular stitches. No drainage was installed routinely.

Bleeding control served as the primary endpoint. As the smallest measurable unit of blood loss represents 25 mL in our routine protocol, this was set as the cut line for minimal blood loss in this study. Secondary endpoints were identified as intra- and postoperative complications as well as the appropriate histopathological outcome in malignancies with regard to free resection margins and local recurrence within a median follow-up of five years.

### 2.2. Statistics

Data were prospectively collected and documented in an Access database (Microsoft Corporation, Redmond, WA, USA). A mathematician (TH) not involved in data collection performed the statistical analyses using R, version 3.4.1 (https://www.r-project.org/). Continuous data are presented as mean ± SD with min–max; categorical data are represented as *n* (%). Differences between groups were assessed using Welch’s two-sample T test for continuous variables and Fisher’s exact test (where applicable) or Pearson’s chi-squared test for categorical variables. A *p* value < 0.05 was considered statistically significant.

## 3. Results

Demographic parameters of patients undergoing SP-MajH and MP-MajH are summarized in Table 1 (Tab 1); procedural parameters are summarized in Table 2 (Tab 2). All major liver resections were able to be performed with the particular laparoscopic technique without converting to open surgery. One patient with simultaneous colorectal resection was converted to facilitate dissection in the narrow pelvis after successful SP hepatectomy. In SP-MajH, the transumbilical approach was used in 14 (41.2%) patients, whereas 19 (55.9%) resections were performed through a right subcostal incision. Additional trocars were delivered in 3/34 (8.8%) of SP-MajH for better exposure of the operating field. Suspending sutures were used in two patients for retraction on the falciform ligament.

With respect to prior surgical interventions, limited and extended SP adhesiolysis was performed in 13 and 6 patients.

Numbers and type of hepatic resections for SP-MajH/MP-MajH were 4/2 right hepatectomies, 6/1 left hepatectomies, 7/5 right posterior bi-segment lateral resections and 17/6 single segmentectomies (Segment 7 or Segment 8). Intraoperative bleeding control during deep parenchymal dissection was achieved by pre-coagulation (Habib 4X) in 22 (64.7%) SP-MajH and 9 (64.3%) MP-MajH. In all other situations, additional thorough preparation with CUSA, bipolar energy, Hemoloc clips and staplers was necessary to ensure safety. Amount of blood loss and number of patients with intraoperative blood loss greater than 25 mL were significantly higher in the MP group. However, the individual amount of blood lost in these patients during SP and MP surgery yielded in mean 1095.5 and 607.7 mL for SP-MajH and MP-MajH, respectively (*p* = 0.56, estimate with 95% CI 487.8 (−387.8 to 1363.4)). It is of note that 63.7% (7/11) of SP-MajH patients with bleeding during the procedure required red blood cell (RBC) packs, whereas only one out of 13 (7.7%) patients with bleeding during MP-MajH was given RBC units (*p* = 0.008, odds ratio (OR) 17.94 (1.59 to 1014.8)). One colon laceration during adhesiolysis accounted for the only intraoperative complication other than bleeding in the SP-MajH group. With regard to concomitant procedures in nine patients, the particular time for liver resection was calculated as mean ± SD 133 ± 53 min in SP-MajH. The surgical approach served as the retrieval site in all patients. The incisional length matched the minimum diameter of the specimen.

Wound closure was documented and evaluated by the surgeon as optimal (*n* = 48), suboptimal (with minor flaws, *n* = 0) or poor (with major flaws, *n* = 0) at the end of SP and MP procedures. Surgical site infections were not observed in any patient.

Postoperative complications classified as Grade 2 or higher according to Dindo-Clavien (DC) [11] were documented in seven (20.6%) and three (21.4%) patients in SP-MajH and MP-MajH, respectively (*p* = 1, estimate with 95% CI 1.05 (0.15 to 5.75)). Types of complications were pleural effusion (*n* = 4, DC 3a), abscess formation (*n* = 1, DC 3a), ascites (*n* = 1, DC 2) and bilioma (*n* = 1, DC 3a) in patients with SP-MajH and pleural effusion (*n* = 2, DC 3a) and acute cholecystitis (*n* = 1, DC 3b) in the MP-MajH group.

Postoperative stay was in mean ± SD 10.6 ± 5.5 days for SP-MajH and 11.6 ± 6.4 days for MP-MajH (*p* = 0.838, estimate with 95% CI −0.9 (−5 to 3.2)); 90-day mortality was zero in all patients.

### Pathology

The underlying diseases are listed in Table 3 (Tab 3). Pathologic assessment yielded specimens without tumor lacerations in all patients with malignant disease. Histology revealed free resection margins in 27 (100%) of 27 specimens and 13 (100%) of 13 specimens in SP-MajH and MP-MajH patients, respectively. During a median oncologic follow-up of 61 and 56 months (SP-MajH and MP-MajH), four (14.8%) and five (38.5%) patients suffered from recurrent diseases (apart from the resection plane or metastatic disease), whereas two patients (7.4% and 15.4%) in either SP-MajH or MP-MajH died during the observation period.

## 4. Discussion

During the past decade, SP minor liver resection has been increasingly seen to make good surgical sense due to its proven benefits of minimal invasiveness and optimal cosmetic outcome [3,4,12,13,14,15]. Unfortunately, the SP concept is bothersome for the surgeon as it involves an uncommon type of triangulation and a limited number of deployed instruments. Bleeding control is crucial and technically demanding in all types of laparoscopic liver surgery as reduced bleeding can contribute to prolonged disease-free survival and overall survival [16]. Therefore, the possible high risks of intraoperative bleeding, longer procedural time and greater personal workload are the feared drawbacks of SP-MajH that make surgeons reluctant to offer this minimized approach technique to their patients. A meta-analysis evaluating patients with SP hepatectomies found a significant reduction in blood loss as compared to conventional laparoscopic liver resection [4]. This finding was confirmed in our study as the number of patients with intraoperative bleeding and the total amount of blood loss were significantly larger in the multitrocar population than in the SP cohort. However, this finding might be misleading: when substantial bleeding occurred in SP-MajH, almost two thirds of these patients required RBC transfusions. When more complex instrument manipulation is required during intraoperative emergencies in SP-MajH, meticulous dissection and hemostasis maneuvers, especially suture techniques, might be hampered. Delivering additional trocars for procedural safety in 8.8% of such interventions did not compensate this disadvantage in the study population. This unfavourable technical characteristic in SP surgery is of even more importance since the procedural difficulty index was significantly higher in MP-MajH in this study. With the intent to alleviate parenchymal transection, inline pre-coagulation by means of radiofrequency [7] did not meet the primary endpoint of sufficient bleeding control as a stand-alone technique in laparoscopic major hepatectomies (SP-MajH and MP-MajH) in about one third of procedures. When dealing with more challenging anatomical situations defined by a significantly higher difficulty index in comparison to minor hepatic resections, pre-coagulation techniques are therefore not regarded as the gold standard in parenchymal transection in minimally invasive major hepatectomy [9].

It is of note that a meta-analysis [17] documented better bleeding control but a higher rate of postoperative abscess formation but not biliary leakage or blood transfusion in the inline pre-coagulation group than for crush–clamp liver resections. The complication rates in SP-MajH and MP-MajH presented here reflect the complexity of the underlying disease and are more than acceptable in comparison to complication rates published for open or laparoscopic major hepatectomies (25.9% and 22.4%) [18]. The meta-analysis by Wang et al. showed no significant difference in terms of procedural time when comparing conventional laparoscopy and SP liver surgery [4]. When considering the fact that about two thirds of all study patients underwent combined procedures, the median operative time of less than three hours and the calculated median time for major hepatectomy of about two hours are comparable to procedural times published for laparoscopic and open liver resections [19,20]. The study presented here is embedded in our SP experience exceeding 5000 procedures. Having performed the first MP laparoscopic major hepatectomy and the first pure SP minor hepatectomy in 2008 [21], we further developed SP-MajH in a group of highly selected patients when overcoming an SP-specific learning curve of more than 1000 performed procedures. In addition to all intraabdominal manipulations, the incisional length allows adequate pathohistologic specimen harvest and an optimal cosmetic result in all patients with SP-MinH or SP-MajH. In MP-MajH, specimen retrieval is performed mostly via a Pfannenstiel incision for reduced wound complication rates and improved function and cosmesis [22]. Our standard of care in major hepatectomies includes an intensive care unit (ICU) treatment for the first two days and an observation at the normal ward for another eight days at least, regardless of an open or laparoscopic approach. This is closely related to national insurance policies and the resulting case-specific reimbursement, hampering any reasonable comparison between the groups. Remarkably, during a five-year follow-up, no wound complication occurred in the entire study population. As the SP concept itself by no means confirms increased hernia rates, we currently aim for a total percentage of 2% late onset hernias in ten years of advanced SP surgery at our department. Due to the heterogeneity of our study collective with regard to tumor entity, it is difficult to assess oncological safety other than to document tumor lacerations, free resection margins and local recurrence. In contrast to non-ablative techniques, it is under debate whether margins extending into the ablation zone should be regarded as R1 resection (which did not occur in any of the study patients). Moreover, none of the patients developed local recurrence at the hepatic resection plane during the follow-up period, which speaks for both the accuracy of the SP technique and the value of inline pre-coagulation as an applicable transection mode. However, the authors are certain that meticulous anatomical preparation in all types of liver surgery with tumors adjacent to vital hepatic pedicles or the vena cava must be performed with instruments capable of more precise manipulation such as CUSA, hydro-jet and crush–clamp in combination with clips, staplers or sutures. The argument for the cost effectiveness (direct cost savings of 27.6% of disposables) enabling inline radiofrequency pre-coagulation is certainly not tenable in patients with SP-MajH when there is a substantial risk of perioperative bleeding. The literature has demonstrated convincingly that perioperative complications turned out to determine the financial burden [23]. It should be noted that certain factors might limit the study. The non-randomized study design and strict patient selection following the aforementioned exclusion criteria should be regarded as a limiting factor before generalizing these results. It must be emphasized that, if the required safety could not be guaranteed with SP, a decision for conventional surgery was made at the discretion of the surgeon. A significantly higher difficulty index in the MP-MajH group and a trend to a longer surgery time might be interpreted as a consequence of this. Hospital stay did not serve as a valid outcome parameter for patient recovery in order to compare groups, as hospital and insurance policies—instead of the patient condition alone—were determining factors in the duration of hospital stay. Quality of life was not assessed in this study, but it has been reported that SP results in better quality of life [11,12] than does conventional surgery. The evaluation of any additional benefit other than a reduction in abdominal wall trauma (shorter skin incisions) in the single-port versus the multiport approach was not scientifically targeted. This includes, but is not limited to, biomarkers, such as circulating tumor cells, circulating nucleic acids, extracellular vesicles and proteins. Targeting these biomarkers might have unravelled differences in some oncological entity more sophistically and represents an interesting future perspective. Emphasizing the calculated overall survival and disease-free survival would have no basis for justification due to the heterogeneity of the study population with malignancies and again was not the aim of this study. Therefore, we did not match open cohorts with the study population.

## 5. Conclusions

Intraoperative bleeding, although not common in minimally invasive liver resection, requires unrestricted immediate manipulation, which might be hampered in SP-MajH. Inline radiofrequency pre-coagulation failed to achieve sufficient hemostasis in laparoscopic major hepatectomies. With sufficient experience in both SP and liver surgery, a low complication rate and good oncologic outcome represented by surrogate parameters in strictly selected patients could be demonstrated in our study. However, SP-MajH should still be considered experimental at this time.

## Figures and Tables

**Table 1:** Demographics.

	SP-MajH	MP-MajH	Estimate with 95% CI	*p* Value
Number (*n*)	34	14		
Female gender (*n*)	13 (38.2%)	6 (42.9%)	1.21 (0.28 to 5.07)	1
Age (years) mean (SD)	63.4 (12.8)	62.4 (15.2)	0.9 (−8.7 to 10.5)	0.964
BMI (kg/m²) mean (SD)	26.4 (3.9)	27.4 (4.9)	−0.9 (−4 to 2.1)	0.61
ASA > 2 (*n*)	19 (55.9%)	4 (28.6%)	0.32 (0.06 to 1.41)	0.117
Liver cirrhosis Child–Pugh A (*n*)	6 (17.6%)	6 (42.9%)	3.4 (0.7 to 17.08)	0.139
Previous surgery (*n*)	22 (64.7%)	9 (64.3%)	0.98 (0.23 to 4.63)	1
Malignant underlying disease	27 (79.4%)	13 (92.9%)	0.30 (0.01 to 2.79)	0.407
Future remnant liver volume (%, SD)	78.6 (14.7)	70.4 (11.7)	8.3 (0.1 to 16.5)	0.042

SP-MajH, single-port major hepatectomies; MP-MajH, multiport major hepatectomies; CI, confidence interval; ASA, American Society of Anesthesiologists.

**Table 2:** Procedural parameters.

	SP-MajH	MP-MajH	Estimate with 95% CI	*p* Value
Surgery time (min) mean (SD)	163.8 (80.3)	208.1 (93.1)	−44.2 (−103.4 to 15)	0.146
Difficulty index mean (SD)	6.6 (1.8)	8.7 (2)	−2.1 (−3.3 to −0.8)	0.004
Blood loss (mL) mean (SD)	354.4 (833.6)	564.3 (745.5)	−209.9 (−713 to 293.3)	0.001
Patients with blood loss > 25 mL (*n*)	11 (32.4%)	13 (92.9%)	25.33 (3.11 to 1195.26)	<0.001
RBC units (*n*)	7 (20.6%)	1 (7.1%)	0.3 (0.01 to 2.79)	0.407
Skin incision (cm) mean (SD)	4.8 (2.1)	5.7 (1.7)	−0.9 (−2.1 to 0.3)	0.027
Maximum specimen size (cm)	10.4 (5.1)	10.5 (4.3)	−0.1 (−3.1 to 2.9)	0.798
Minimum specimen size (cm)	5.2 (2.8)	5.1 (2.5)	0 (−1.7 to 1.7)	0.657

SP-MajH, single-port major hepatectomies; MP-MajH, multiport major hepatectomies; CI, confidence interval; RBC, red blood cell.

**Table 3:** Underlying diseases.

	SP-MajH	MP-MajH
Benign diseases		
Giant hemangioma	5	-
Adenoma	-	1
Abscess formation	2	-
Malignant diseases		
● Primary liver tumors		
Hepatocellular carcinoma	8	7
Cholangiocellular carcinoma	1	-
● Liver metastases		
Colorectal cancer	8	6
Neuroendocrine tumors	4	-
Pancreatic cancer	4	-
Breast cancer	1	-
Ovarian cancer	1	-

## Data Availability

The data presented in this study are available on request from the corresponding author. The data are not publicly available due to the hospital’s privacy policy.
